# Zinc hybrid sintering for printed transient sensors and wireless electronics

**DOI:** 10.1038/s41528-023-00249-0

**Published:** 2023-03-14

**Authors:** N. Fumeaux, D. Briand

**Affiliations:** Soft Transducers Laboratory, Ecole Polytechnique Fédérale de Lausanne (EPFL), Rue de la Maladière 71b, CH-2000 Neuchâtel, Switzerland

**Keywords:** Electrical and electronic engineering, Materials for devices, Design, synthesis and processing, Biomedical materials

## Abstract

Transient electronics offer a promising solution for reducing electronic waste and for use in implantable bioelectronics, yet their fabrication remains challenging. We report on a scalable method that synergistically combines chemical and photonic mechanisms to sinter printed Zn microparticles. Following reduction of the oxide layer using an acidic solution, zinc particles are agglomerated into a continuous layer using a flash lamp annealing treatment. The resulting sintered Zn patterns exhibit electrical conductivity values as high as 5.62 × 10^6^ S m^−1^. The electrical conductivity and durability of the printed zinc traces enable the fabrication of biodegradable sensors and LC circuits: temperature, strain, and chipless wireless force sensors, and radio-frequency inductive coils for remote powering. The process allows for reduced photonic energy to be delivered to the substrate and is compatible with temperature-sensitive polymeric and cellulosic substrates, enabling new avenues for the additive manufacturing of biodegradable electronics and transient implants.

## Introduction

Transient electronics, i.e. electronic components and devices that fully degrade in a given environment without generating harmful byproducts^1,2^, show potential in reducing electronic waste^3–5^, and enable novel bioresorbable implants, eliminating the need for a re-operation^6–8^. Different materials such as dissolvable metals^9–11^, degradable semi-conductors^2,12,13^, substrates and dielectrics^14–18^ have been proposed. These developments have led to various devices being demonstrated, including batteries^19–21^, heaters^22,23^, transistors^24,25^, energy harvesters^26,27^, as well as pressure^28–30^, strain^31^, and temperature sensors^32^. Most of these functional biodegradable components and devices rely on microfabrication techniques stemming from the semiconductor industry, and the use of shadow-mask techniques^26^ or transfer printing^33^ to circumvent the difficulty to pattern functional layers directly onto temperature and solvent-sensitive biodegradable substrates^8^. As an alternative, additive manufacturing techniques have shown promise for the fabrication of flexible transient electronics, especially in domains where large area, cost-effective and low-waste manufacturing is desired. Digital additive manufacturing also presents the advantage of allowing freeform printing on 3D curvilinear surfaces^34^ and seamless integration of multi-sensing paradigms^35^. This expands the design possibilities to customizable, deformable or highly conformal sensor networks, which can be self- or remotely powered^36^. These manufacturing methods have the potential to open new possibilities for the fabrication of functional degradable electronic devices, but require joint optimization of printable ink formulation, deposition processes, and post-treatment methods. After deposition of a transient metal micro- or nanoparticles ink, the conductivity is in general very low or non-existent and a sintering step, i.e., a densification of the material without melting to the point of liquefaction, is required^36^. This is especially challenging due to the reactivity of these materials and their tendency to naturally form an oxide layer in air. Only few approaches have been proposed to achieve sintering of printed biodegradable electrically conductive layers. These approaches focus on zinc because of its low melting point and owing to its widespread availability in micro- and nanoparticle forms at affordable prices^11,37–42^. Methods applied so far to sinter zinc particles can be classified into two main categories: photonic and electrochemical. Photonic methods are based on the use of high-power lasers or lamps^37,39,40,43^ to selectively heat the metallic layer while minimizing the interaction with the substrate. They are usually restricted by the high-melting (*T*_m_ = 1950 °C) native oxide layer of the Zn particles, which prevents efficient particle agglomeration by maintaining the metallic zinc in a solid shell^40^. Even by applying sintering pulse intensities superior to 10 J cm^−2^, the electrical conductivity reached remains limited^39^. Electrochemical sintering of zinc is a room-temperature method that leverages the interaction between acetic acid^11,42^, or analogues such as propionic acid^41^, to convert the native oxide layer to Zn ions. The metal ions redeposit between the particles, thereby forming a conductive path. Electrochemical sintering usually yields lower conductivity values (in the order of 1–3 × 10^5^ S m^−1^) due to the minimal bridging achieved between the particles and the presence of residual binder in the layer. Finally, a recurrent challenge that arises when fabricating complex flexible devices with transient metals is that their reactivity precludes the use of multiple post-processing steps which may damage the metal traces. As a consequence, robust processes need to be developed for the additive manufacturing of eco- or bioresorbable metal traces, compatible with post-processing steps and yielding stable devices with a sufficient time of operation.

In this work, we present a hybrid method for the scalable and additive manufacturing of highly conductive transient metallic zinc traces. After deposition by printing, the native oxide shell of zinc microparticles is reduced by means of an acetic acid solution. The reducing agent is delivered via an optimized spray coating process, with the aim of mitigating the downsides of previously presented dispensing methods^42^. Flash lamp annealing (also referred to as photonic sintering) is used to further sinter the metallic patterns. This approach permits to reach unparalleled electrical conductivities for printed transient metal, up to 5.62 × 10^6^ S m^−1^, only about three times less than the conductivity of bulk zinc (16.6 × 10^6^ S m^−1^). As an added benefit, the energy delivered to the sample is considerably lower than in previous studies concerning zinc photonic sintering^39,40^. This allows for increased compatibility with lower thermal budget substrates. The mechanical flexibility of the printed traces as well as their stability in environments relevant to their operation are demonstrated. The fabrication of multilayer devices requiring further curing or deposition steps is enabled by the robustness of the transient metal traces. A set of printed physical sensors, including resistive strain and temperature sensors, as well as a wireless capacitive force and pressure sensor are reported. The process presented in this work advantageously leverages chemical and physical mechanisms to obtain highly conductive (comparable with heat-cured copper or silver inks^44^) transient metal traces. These advances unlock new avenues for environmentally friendly internet-of-things components and bioresorbable electronic implants fabricated by additive manufacturing.

## Results and Discussion

### Hybrid sintering of printed zinc films

**Fig. 1:**
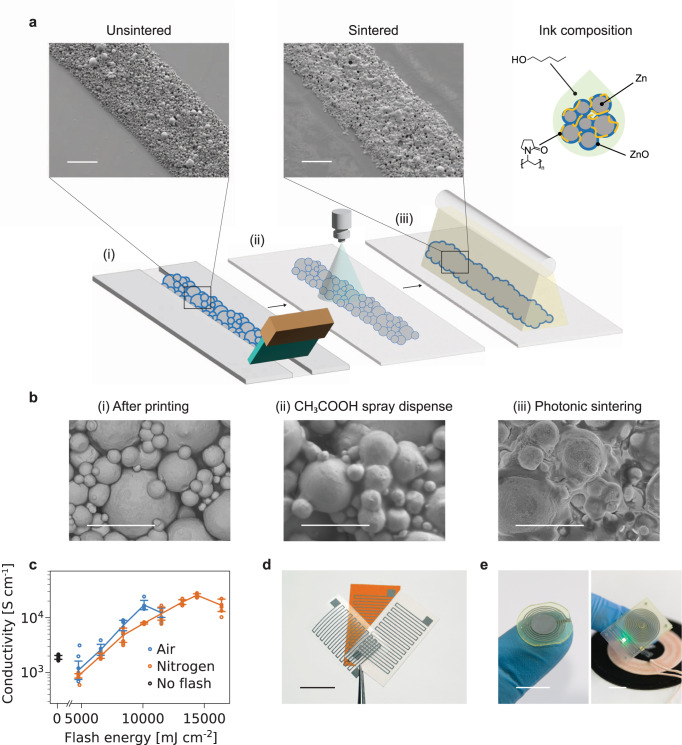
Description of the hybrid zinc sintering process. **a** Illustration of the steps of the hybrid sintering process (**i**) deposition by printing (**ii**) acetic acid spray coating (**iii**) photonic sintering. Scale bars are 100 μm. **b** Microstructure of the Zn by SEM after each processing step. Scale bars are 10 μm. **c** Measured conductivity (with standard deviations) of Zn traces at different pulse energies and under air or nitrogen atmosphere. **d** Printed Zn resistors on polyimide, paper, and polylactic acid substrates. Scale bar is 10 mm. **e** (left) Bioresorbable radio-frequency chipless pressure sensor (right) wirelessly powered LED circuit based on a Zn secondary coil on a degradable substrate. Scale bars are 10 mm.

The sintering method that is presented here is compatible with commercially available microparticles obtained through a powder atomization process, and no further step such as ball milling is necessary. These zinc microparticles (2 µm average diameter) are mixed with polyvinylpyrrolidone (PVP) as a binder and pentanol as a solvent to form the printable ink. The hybrid sintering relies on a two-step process as follows (Fig. 1a): (i) acetic acid treatment by spray coating, to reduce the oxide layer and (ii) photonic sintering, where energy in the form of high intensity pulsed light is delivered to achieve particle agglomeration into a continuous metallic trace. SEM imaging (Fig. 1b) is used to show the evolution of the microstructure of the zinc printed layer between the processing steps. Separated particles can be clearly observed just after printing and solvent drying. The acid sintering only slightly modifies the microstructure, with conductive bridges formed between particles in the order of a few hundreds of nanometers, as observed in previous work^11^. Conversely, after the application of pulsed light, microparticles are shown to have fused and aggregated into a continuous layer. Preliminary experiments conducted on printed zinc resistors on polyimide show that photonic sintering delivers an improvement in conductivity by more than one order of magnitude compared to solely using electrochemical sintering (Fig. 1c), whether conducted under air or nitrogen atmosphere. The synergistic interaction between those treatments, i.e., the removal of the oxide layer and the formation of bridges between the particles considerably enhancing the effect of the photonic sintering, allows outstanding conductivity for printed biodegradable metallic films. The results in air and N_2_ atmospheres are comparable, although a higher conductivity (∼50% higher) is reached in an inert atmosphere. This method of sintering is compatible with various substrates used in standard, paper, and degradable electronics (Fig. 1d). This versatility as well as the remarkable electric conductivity that is achieved with the hybrid sintering process makes it suitable for the fabrication of degradable radio-frequency devices. Notably, we demonstrate a fully printed bioresorbable LC circuits applied to chipless pressure sensing as well as a degradable wireless power receiver (Fig. 1e).

### Optimization of sintering parameters

**Fig. 2:**
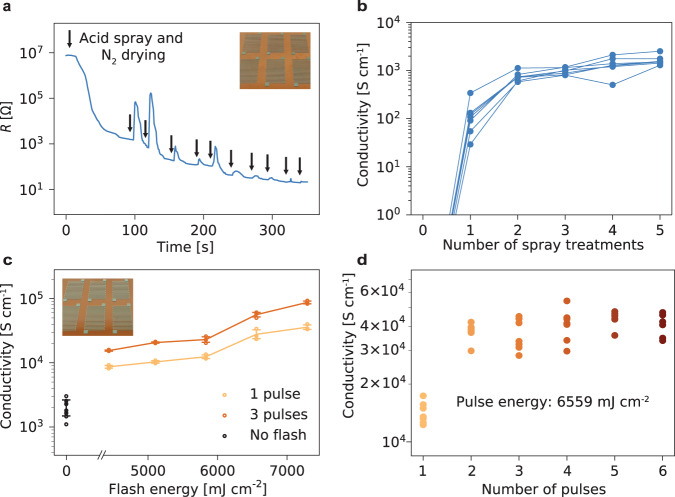
Study of the sintering process on screen-printed zinc layers. **a** Real-time measurements of the resistance of a Zn line under consecutive acetic acid spray coatings and nitrogen drying. **b** Conductivity of Zn resistors (with standard deviations) on polyimide after electrochemical treatment cycles. **c** Conductivities of Zn resistors treated with electrochemical and photonic sintering, for 1 and 3 pulses of various energies. **d** Influence of the numbers of pulses on the conductivity of sintered Zn for a given pulse energy of 6559 mJ cm^−2^ (*n* = 7 per pulse).

A first study on the process parameters used for the electrochemical and photonic sintering steps was performed on resistors printed on polyimide substrates. Firstly, we examined the electrochemical treatment of the printed layers by acetic acid spray coating. The use of spray coating relies on limited and uniform acid deposition to avoid the partial dissolution of the unsintered zinc patterns, which is a known issue when using a drop coating process to deliver the acidic solution^41^. Moreover, the electrochemical sintering process occurs in a short timeframe (less than one minute)^11^. We observed that fast drying (on a hot plate or by application of a stream of N_2_) was critical to avoid damage to the metal traces from prolonged exposure to the aqueous solution. As a consequence, several coatings and drying cycles must be applied to reach a plateau conductivity in the order of 1980 S cm^−1^. As shown in Fig. 2a, the improvement in conductivity can be obtained only by alternating the application of acetic acid with a drying step in N_2_. As can be seen, the resistance initially increases after dispensing, as the porous track is soaked with the acid solution, and nitrogen drying causes the solution to dry and the Zn ions to depose between the particles. A saturation in conductivity is reached after the application of 5 to 6 of these cycles (Fig. 2b). The concentration of the PVP binder in the inks has an influence not only on its printability but also on the conductivity achieved following the electrochemical sintering step, as observed previously^41^. Therefore, keeping the solvent to solid material ratio constant, the effect of the chain length and the concentration of PVP with respect to the amount of metallic powder on the conductivity of the films was evaluated (Supplementary Fig. 1). The chain length was shown to have minimal influence on the conductivity in the chosen range. On the contrary, the concentration of binder was shown to influence the obtained electrical conductivity. A ratio of 0.04 g of PVP per g of Zn was deemed to be optimal and was chosen for further inks formulation. The conductivity obtained with the spray treatment is comparable with results previously obtained with electrochemical methods in the literature^11,41,42^. The conductivity reaching a plateau serves as a validation of the dissolution of the oxide layer, as no more oxide is converted to metallic zinc. This is critical for achieving efficient particle agglomeration and high conductivity by photonic sintering. The cycling of the acid treatment could be seen as a limiting factor for the scalability of the process. However, it is likely that the dispensing could be optimized for throughput outside of a research setting. Moreover, the reducing agent may be incorporated to the ink in future work, to circumvent the necessity of spray dispensing altogether.

The second step of the hybrid process, photonic sintering, was studied varying the pulse energy and the number of pulses delivered (Fig. 2c). The study was conducted under a nitrogen atmosphere, to avoid reoxidation effects, notably when studying the effect of multiple photonic pulses. Moreover, as shown above, sintering in a nitrogen atmosphere leads to higher conductivities, which we hypothesize arise from the fact that the inert atmosphere allows to limit zinc oxidation, which may hinder conductivity^45^. As can be seen in Fig. 1c, higher pulse energies appear to be necessary to reach maximal conductivity in the case where sintering is conducted under an inert atmosphere. This is however likely due to the increased distance from the lamp caused by the sealed chamber used, therefore decreasing the effective energy delivered to the samples. The energy delivered in one pulse of photonic sintering depends on the voltage supplied to the xenon lamp and the irradiance profiles of the pulses applied are given in Supplementary Fig. 2. The timing between the spray coating and the photonic sintering steps is of importance, however, a precise study of its influence is difficult as it would depend on the exact drying conditions of the acid (atmosphere, flow), as well as on the morphology of the printed layer, notably dimensions and porosity. We observed that if photonic sintering was conducted after more than one hour of drying in ambient air after the acid application, the electrical conductivities obtained started to decline, as a result of the oxide layer reforming. We hypothesize that residual acidic solution in the metallic microparticles layer is critical to avoid re-oxidation before the photonic sintering step. Applying photonic sintering generally increases the electrical conductivity of the zinc traces by one to two orders of magnitude compared to samples that underwent only electrochemical sintering, and the increase is more marked when several light pulses are delivered (Fig. 2c). The conductivities that were measured reached average values as high as 5.62 × 10^4^ S cm^−1^, when 3 pulses of 6559 mJ cm^−2^ were delivered. To our knowledge, this is the highest conductivity value obtained for printed zinc layers (Supplementary Fig. 3). The values obtained for the highest studied pulse energy (7299 mJ cm^−2^) are not considered as this level of energy was causing partial destruction of the resistors and inferior yield. At this pulse energy, a yield of 25% was observed versus near 100% for lower energies. Indeed, increasing the pulse energy will lead to increased sintering as the metal layer approaches its melting point^46^, but an energy that is too high will lead to considerable melting and flowing of the metal, which causes interruptions in the conductive line. Conductivity values for samples sintered above 7300 mJ cm^−2^ could not be measured due to damages and cracks occurring in the layers at these high levels of energy, as observed through optical microscopy. Using several light pulses was observed to improve conductivity for the same pulse energy, however, the improvements started to plateau after two to three light pulses (Fig. 2d). This behavior could be explained by an equilibrium being reached between the damage caused to the tracks by further energy being delivered and the improved particle cohesion with more pulses. The variability in conductivity between samples is in line with previous studies on photonic sintering^39^, and could be further reduced by using smaller metal particles or a more controlled acid dispensing technique. When observing the cross-section of the sintered Zn thick films under a scanning electron microscope, the particles display a degree of agglomeration that varies across a gradient, with the first 5 to 10 µm forming a cohesive layer independent of thickness (Supplementary Fig. 4). We note that a more than twice higher conductivity is obtained for screen printed lines (17 µm average thickness) compared to stencil printed lines (41 µm thickness, see Fig. 1c), which was measured to be 2.55 × 10^4^ S cm^−1^. This may be due to enhanced sintering on the uppermost zinc layer; however, the process is demonstrated to be amenable to varying pattern thicknesses. As can be expected, the energy required for optimal sintering of the thinner layers is lower. In order to verify that the resolution of the lines is maintained throughout the process, the profile of the lines was compared between each treatment step (Supplementary Fig. 4) and no significant difference in area was observed. Moreover, good adhesion between the sintered zinc layer and polyimide, paper, and PLA substrates was observed with a standard peeling scotch test (Supplementary Fig. 5). In order for this method to be usable for films printed with other deposition methods, it would require to be compatible with the use of nanoparticles, as methods such as aerosol-jet printing, inkjet printing or direct ink writing require smaller particle sizes. We hypothesize our method to be compatible with the processing of nanoparticles films, in particular because photonic methods are amenable to the sintering of nanoparticles^39,40^, as the reduction in particle size comes with a reduction of the melting point, and, as a consequence, of the required energy to attain efficient sintering. Preliminary tests with 500 nm Zn particles were conducted and conductivities reaching 3.3 × 10^6^ S m^−1^ were obtained, with the same ink formulation as the one aforementioned and using a lower pulse energy (5.1 J cm^−2^).

### Flexibility and stability of fully degradable zinc traces

**Fig. 3:**
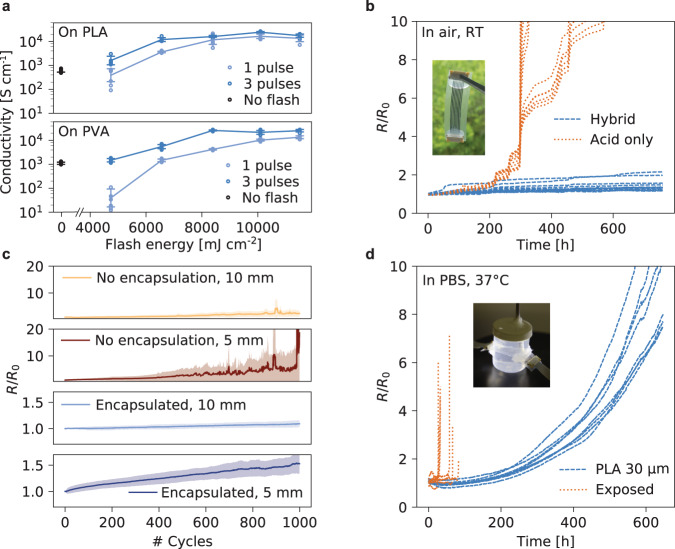
Hybrid sintering of Zn and durability of the traces on transient substrates. **a** Electrical conductivity (with standard deviations) for sintered Zn traces as a function of energy and number of photonic pulses, on PVA and PLA. **b** Evolution of the resistance for unencapsulated Zn samples (*n* = 14 per condition, 40 × 0.3 mm) stored at ambient conditions, as a function of the sintering protocol. **c** Change in resistance for sintered Zn lines on PLA (*n* = 5 per condition, 50 × 0.5 mm, with a ~40 µm thickness), for radii of 5 mm and 10 mm, with and without PLA encapsulation. **d** Evolution of the resistance for encapsulated and unencapsulated sintered Zn tracks (*n* = 8 per condition, 40 × 0.3 mm) in PBS at 37 °C.

Importantly, to take full advantage of this process to sinter transient metal, it needs to be compatible with temperature- and solvent-sensitive bioresorbable and biodegradable substrates. This is possible with the two-step sintering method presented here, thanks to the use of flash sintering, which allows to selectively heat the metal tracks with minimal energy being directly transferred to the transparent polymeric substrates^47^ as well as cellulosic substrates^48^. Moreover, the use of spray-coating to dispense the acid is also beneficial by limiting the amount of aqueous solution on the water-sensitive substrate, compared to methods such as drop-casting for instance^11^. The hybrid method we introduce here is compatible with flexible paper substrates, which are of interest in the context of biodegradable and eco-friendly electronics^49^. Printed Zn traces processed on paper reach an average electrical conductivity of 2.00 × 10^4^ S cm^−1^ (Supplementary Fig. 6). Furthermore, we study the sintering of zinc on thin layers of bioresorbable polymers. The results of zinc hybrid sintering poly(vinyl acetate) (PVA) and polylactic acid (PLA) are shown in Fig. 3a. Similar maximal mean conductivities of 2.61 and 2.43 × 10^4^ S cm^−1^ are obtained, respectively for PVA and PLA, following three light pulses with an energy of 8390 mJ cm^−2^. Remarkably, these high values in electrical conductivities are similar to the results achieved on polyimide. The slight reduction in terms of conductivity that is observed may be due to a degradation of the interface substrate-zinc layer, which could cause deformation and cracking of the zinc resistors.

Ideally, transient electronic devices would display stable function during their lifetime and rapid degradation upon the introduction of a specific stimulus or at the end of the desired lifecycle^50^.

Experiments for zinc samples made on PLA were conducted to assess the electrical durability of the traces in conditions reproducing the operating environments for applications such as biodegradable electronics (e.g., for smart packaging or environmental monitoring) and bioresorbable electronics (e.g., smart implants for post-surgical monitoring). In the case of samples that were only sintered electrochemically, the conductivity of the Zn traces changes in a non-linear way after a week, and a tenfold increase in resistance is already attained after 300 to 500 h of exposure to ambient air. Conversely, the Zn traces that were fabricated with hybrid sintering and without encapsulation display a much more stable electrical conductivity for the period of measurement (31 days) with the resistance increasing by a factor of 1.4 only, in air at ambient conditions (Fig. 3b). This difference can be explained by the increased aggregation of particles in the case of hybrid sintering, which reduces the impact of the reoxidation process, resulting in a lower relative loss of conductivity. For Zn traces that were only electrochemically sintered, the smaller bridges formed between particles are converted back to ZnO, thereby reducing the conductivity of the layer. The proposed mechanism is illustrated in Supplementary Fig. 7. When aiming for real-world electronic applications, encapsulation is often needed and hybrid sintered Zn samples that were encapsulated with blade-cast PLA displayed an average loss of conductivity of 12% after 31 days at ambient conditions (Supplementary Fig. 8). With the aim to study the usability of the process in the fabrication of transient implantable electronics, the durability of hybrid sintered Zn lines was assessed in a sealed vial containing phosphate-buffered solution (PBS) at 37 °C, which emulates in vivo experiments (Fig. 3d). The conductivity of unencapsulated zinc traces degrades by an order of magnitude within 46.6 h on average, as they disintegrate rapidly in an aqueous medium^8,9^. With a 30 μm encapsulation considerably reducing water permeation, the resistance gradually increases, and it takes on average 28 days to reach a loss of conductivity of 90%. In line with previous work, the degradation of transient metal is rapid in bodily fluids and the choice of the encapsulation with respect to its water permeability and degradation speed is key to maintain function for the amount of time required by specific applications^17^. It is important to note that the degradation in conductivity in the porous metallic layer does not directly correspond to physical degradation. The rate of zinc corrosion in various environments has been characterized in other studies^9,28^. Considering a layer of zinc with a thickness of 40 µm without encapsulation and a constant mass loss rate per unit area rate in PBS at 37 °C of 0.025 mg cm^−2^ h^−1^ ^28^. the time for the full resorption of the zinc layer could be estimated to 48 days.

In order to fabricate reliable transient devices, the mechanical flexibility of the metallic interconnects is an important feature. In the case of inks that are only chemically sintered at room temperature, the presence of binder in the layer may contribute to the flexibility of the conductive patterns by acting as a plasticizer^41^. However, here, the flash sintering step likely vaporizes the PVP binder^51^. The hybrid sintered Zn tracks can be bent down to a radius of 3 mm without loss in conductivity (Supplementary Fig. 9). The mechanical flexibility of the printed Zn lines, with and without encapsulation, was also evaluated by applying repeated bending cycles (Fig. 3c). As expected, encapsulated samples exhibit better resilience to repeated bending, as the neutral plane is shifted closer to the metal line, and the polymeric encapsulation likely reinforces the porous metallic layer^11^. Indeed, in the case of encapsulated samples bent at a radius of 10 mm, the resistance increases by 9% after 1000 cycles at 0.5 Hz, as shown in Fig. 3c, while the resistance of unencapsulated samples more than doubles after 1000 bending cycles. The increase in resistance under fatigue bending is attributed to the formation and propagation of micro-cracks in the metallic line^52^.

### Resistive and capacitive wireless transient sensors

**Fig. 4:**
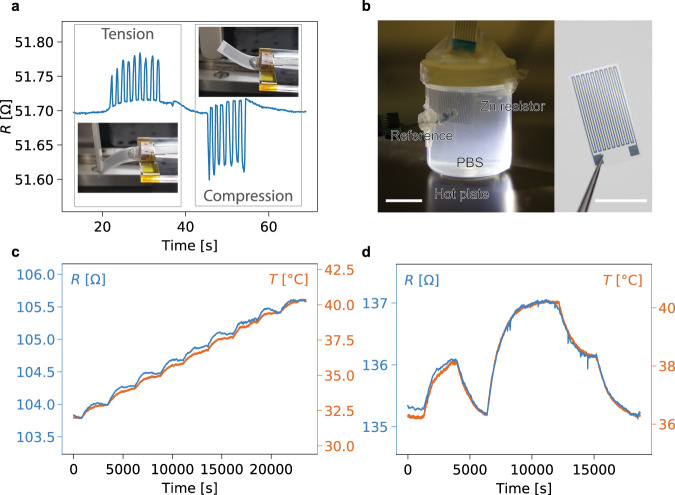
Transient resistive physical sensors fabricated from printed Zn encapsulated in PLA (R_0_ = 89.6 ± 56.4 Ω, *n* = 4). **a** Zn strain sensor submitted to compressive and tensile cycles (insets). **b** Zn temperature sensor in a vial of PBS, with a reference temperature sensor. Scale bars are 10 mm. **c** Zn resistance change (blue) following an increasing temperature in PBS (orange). **d** Zn resistance change (blue) with an arbitrary change in PBS temperature (orange) around physiological temperatures.

The enhanced electrical conductivity, electrical stability, and flexibility, reached with the hybrid sintering method of the printed zinc films can be exploited to fabricate simple transient resistive sensors on PLA. Figure 4a shows a resistive strain sensor undergoing repeated bending in compression and tension. The resistance value varies by approximatively ± 0.2% and returns to the baseline value upon release of the sensor. The temperature behavior of the zinc degradable resistors was studied with the aim of fabricating resistance thermometers. The change in resistance for temperatures up to 60 °C was measured (Supplementary Fig. 10). Zn lines that underwent hybrid sintering show a stable behavior under temperature changes, as opposed to electrochemically sintered traces. A linear relationship between the changes in resistance and temperature was observed for Zn resistors. The temperature coefficient of resistance (TCR) of the printed zinc after hybrid sintering was calculated to be 0.00316 °C^−1^, which is close to the TCR of bulk zinc^53^ (0.00385 °C^−1^) as well as that of standard platinum-based temperature sensors^54^, further validating the efficiency of the hybrid sintering method. Figure 4b shows such a temperature sensor operating in PBS solution placed on a hotplate, with the temperature in the solution controlled with the aid of a commercial temperature sensor (Sensirion). The change in resistance of the two sensors was measured for variations of temperature around 37 °C. The resistance varies linearly with the temperature and the sensors are sensitive enough to detect small changes in temperature within the physiological range (Fig. 4c). The fully degradable zinc-based resistors demonstrate good tracking of the temperature in agreement with the reference data (Fig. 4d). Although the proposed resistive sensors are sensitive to various physical inputs, strategies have been proposed to address this challenge^55^. In our case, different designs could be used to obtain different sensitivities to temperature and strain for instance^56^ (e.g., meander and serpentines), with the aim to fabricate a fully bioresorbable multi-sensor platform.

**Fig. 5:**
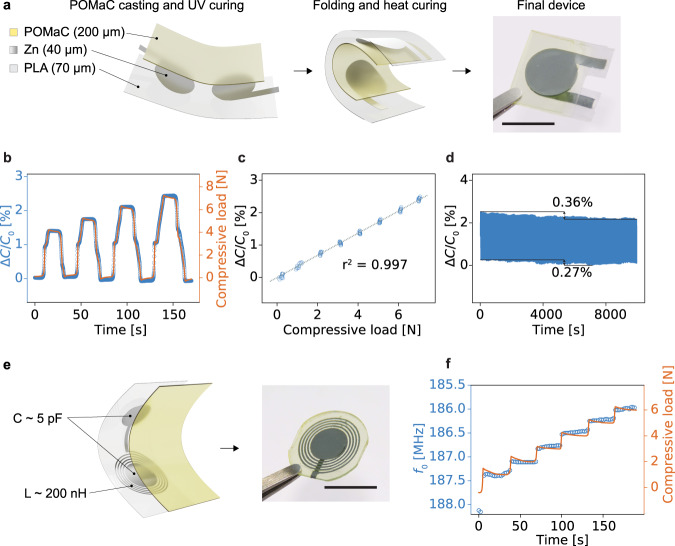
Bioresorbable capacitive sensors based on zinc hybrid sintering and soft elastomer POMaC. **a** Schematic of the fabrication and picture of the Zn-POMaC capacitive sensor. **b** Capacitive response of the pressure sensor under compressive load. Scale bar is 10 mm. **c** Relative change in capacitance over 10 cycles for forces up to 7 N. **d** Cycling of a capacitive sensor for 10000 s (0–4 N, 40 s period). **e** Schematic and picture of the Zn-based RLC sensing circuit. Scale bar is 10 mm. **f** Response of the wireless pressure sensor in resonant frequency change under compressive load.

Finally, we demonstrate that the zinc layers as sintered here are compatible with the fabrication of advanced devices requiring further curing and assembly steps, such as multilayer soft wireless sensors. The hybrid sintering of the zinc layer is implemented to realize original device designs, such as those shown in Fig. 5. Transient capacitive pressure sensors relying on a soft bioresorbable elastomer poly(octamethylene maleate (anhydride) citrate) (POMaC)^57^ as a dielectric layer are presented. The POMaC pre-polymer was synthesized as in previous works^30^ and an exemplary ^1^H-NMR spectrum is shown in Supplementary Fig. 11. POMaC is a soft elastomer which has been used in past publications as an transient encapsulation, substrate or structural material^30,31,58,59^, owing to its stretchability and ease of processing by ultraviolet (UV) photopolymerization. The design of the uniaxial pressure sensor is based on a parallel plate capacitor architecture, with the POMaC layer acting as a deformable dielectric, which compresses under pressure leading to an increase in capacitance. A facile fabrication process was developed, leveraging the dual cross-linking mechanism of POMaC and the flexibility of the substrate and printed layers. As shown in Fig. 5a, the metallic plates and contact pads for the capacitive sensors were printed and treated on PLA, with the POMaC pre-polymer deposited on top by blade casting. The pre-polymer was cured by UV photopolymerization, yielding a solid but tacky polymeric layer. The assembly was then folded with the POMaC layer on the inside and further allowed to cure and bond at 80 °C for 48 h. The Young’s modulus of POMaC after UV and heat curing was measured to be 1 ± 0.2 MPa and its relative permittivity ϵ_r_ was estimated to be 6.9 ± 0.4. The sensors were tested for forces up to 10 N and the response in capacitance followed the applied force closely and maintained a stable baseline (Fig. 5b). The capacitive devices shown here had an initial capacitance of 15.5 ± 3.4 pF (*n* = 5) and the change in capacitance was linear (*R*^2^ = 0.997) with the force applied, with a sensitivity of 71.4 fF N^−1^ (Fig. 5c). This corresponds to a relative sensitivity of 0.00035 kPa^−1^, which is on the lower end of the state of the art for bioresorbable pressure sensors^60^. However, these sensors rely on micro-/nanostructured dielectrics that could be implemented in future developments to reach higher sensitivity. The device was also evaluated under cycling conditions for 2500 cycles with a duration of 40 s and an amplitude of 4 N (Fig. 5d). The maximal value of capacitance recorded during the whole cycling period varied by 0.36 % and the baseline by 0.27 %. The change of performance of the sensors over longer periods of time would depend on the evolution of the viscoelastic properties of the POMaC dielectric^61^. This would in turn depend on the encapsulation of the sensor and the conditions of operation^62^, and more research on the degradation of bioresorbable elastomers is needed to systematically evaluate the long-term performance of the sensors. Bioresorbable sensors are envisioned for applications in post-operative monitoring or regeneration, and while transient metallic wires and connections can be used to interface them^63^, wireless powering and communication are a desirable option to minimize invasiveness. Therefore, a similar design with a zinc coil in series was fabricated (Fig. 5e), relying on the same folding process. The sensing relies on the shift in the resonant frequency of the series RLC circuit formed by the Zn-POMaC capacitive element characterized above and the Zn antenna. This allows to create a fully bioresorbable chipless circuit to sense forces and pressures, taking advantage of the higher conductivity obtained with the hybrid sintering process. Supplementary Fig. 12 shows the return loss (*S*_11_) of typical devices as measured with a custom-made silver antenna on FR4, and they have a resonant frequency of 154.7 ± 3.3 MHz. The response is comparable to the soft capacitors shown above, with the resonant frequency shift (320 kHz N^−1^) being proportional to the applied force (Fig. 5f). A similar process was applied to manufacture the degradable wireless power receiver shown in Fig. 1e. In order to demonstrate the possibility to integrate silicon-based dies with the degradable electronic receiver and board, an LED and a capacitor were soldered using a room-temperature cure degradable Zn-based paste. The double-sided Zn coil separated with a POMaC layer was measured to have an inductance of 3.2 µH and the LED could be powered at a distance of up to approximately 60 mm. A similar setup shown in previous research was used to drive the primary coil^64^, and the peak-to-peak voltage elicited in the biodegradable receiver as a function of the distance to the primary coil is shown in Supplementary Fig. 13.

In summary, the hybrid approach for transient metal sintering presented in this work addresses several challenges related to the fabrication of degradable electronic devices on temperature and water-sensitive substrates. The combination of two complementary sintering approaches, namely electrochemical and photonic, allows to overcome the disadvantages of each method and achieve outstanding particle agglomeration. Indeed, hybrid sintering enables the highest electrical conductivity shown thus far for printed transient metal, as well as a high temperature coefficient of resistance, owing to the enhanced particle cohesion. The reliability of the sintering process in combination with the stability of the printed zinc enable the realization of a wide range of original and both fully printed and biodegradable physical sensors, notably transient temperature and wireless pressure sensors, as well as RF coils and antennas. The study presented here paves the way to cost-efficient and eco-friendly printed sensors and transducers for applications such as supply-chain or environmental monitoring as well as transient implants processed using digital additive manufacturing. This could enable, for instance, the freeform printing and curing of highly conformable and customizable degradable electronics^43^. The digital additive manufacturing of systems such as deformable 3D printed sensor networks or self-powered sustainable electronics^65^ present interesting new directions for the work presented here.

## Methods

### Preparation of Zn-based inks

Zn microparticles (2 µm average diameter, Sigma Aldrich), polyvinylpyrrolidone (Sigma Aldrich, Mw = 360 K or 2000 K) and Pentanol (Sigma Aldrich) were mixed at varying weight ratios (25:1:5 for the final formulation). The ink was homogenized with a planetary mixer (Thinky ARE-250) at 300 rpm for 30 min. It was stored at 4 °C and brought to room temperature and homogenized at 300 rpm for 10 min before printing.

### Preparation of substrates and encapsulation layer

The substrates used in this study are polyimide (DuPont, Kapton® 125 µm), biodegradable paper (Arjo-Wiggins Powercoat XD, 200 µm), polylactic acid (Ingeo™ Biopolymer 4032D) and polyvinyl alcohol (Sigma-Aldrich, Mowiol 4–88). For the preparation of PLA films, pellets were dissolved in 1,4 dioxane (Sigma-Aldrich) overnight at 50 °C under stirring to obtain a 15 wt% solution. Films of PLA were prepared by blade-casting the solution on a standard 4-inch single-side polished silicon wafer at a speed of 2 mm s^−1^ with a gap of 1000 µm to obtain films of 70 µm in thickness, after overnight drying. 25 wt% solutions of PVA were prepared by dissolving PVA for 3 h in DI water under stirring. The PVA films were cast as described above, with a gap of 600 µm to obtain films with a thickness of 75 µm. Before printing, all substrates, except the paper, were first activated with an oxygen plasma treatment for 60 s at 200 W at 40 kHz (Diener). For the encapsulation of the sintered Zn traces, the same PLA solution was blade-casted with a gap of 500 µm, to yield an encapsulation of approximately 35 µm in thickness.

### Printing of Zn patterns

For the stencil printing, one-sided polyethylene adhesive tape (Nexus G20, 80 µm) was cut with the desired patterns with a CO_2_ laser (Trotec Speedy300). The Zn ink was applied with a silicone squeegee and left to dry for 1 h, after which the stencil was peeled off from the substrate. For screen printing, custom stainless steel meshes (Serilith) were used, and the ink was applied through the stencil with a silicone squeegee, at a distance of 500 µm from the substrate. The traces were left to dry for one hour before further processing.

### Treatment of Zn patterns

Acetic acid (10 vol% in de-ionized water) was spray-coated on the Zn traces using an airbrush (Harder & Steenbeck, 0.2 mm nozzle, at a distance of approximatively 10 cm from the substrate) supplied by 2 bar N_2_ pressure, followed by 2 min drying under nitrogen. This spray-coating/drying cycle was repeated a total of 5 times. In the case where the process atmosphere was controlled, the samples were placed in a custom chamber that was purged with nitrogen for 45 s. The photonic sintering treatment (Novacentrix PulseForge 1200) comprised pulse energies ranging from 3107 to 12867 mJ cm^−2^ and between 1 and 6 pulses were delivered.

### Material characterization

The thickness and morphology of the printed Zn patterns were characterized using a laser scanning confocal microscope (Keyence VK-X1000). The microstructure of the lines with different treatments was inspected by SEM (JEOL JSM-7500TFE). For SEM inspection of sintered Zn thick films, printed patterns were cleaved after rapid cooling in liquid nitrogen. The resistance of the Zn lines was measured using a tabletop multimeter (Keysight 34401 A). To avoid contact resistance effects, 4-wire measurements were performed with custom Kelvin probes. For bending tests, Zn lines were stencil-printed, sintered, and encapsulated as described above if required, the outlines of the samples were laser-cut and the samples were peeled off from the silicon wafer. A custom setup consisting of clamps and a linear motor was used. The adhesion test was performed according to ASTM F1842-15, on 2 × 2 mm^2^ sintered Zn squares on polyimide, paper, and PLA. For the degradation tests, Zn lines were fabricated as described above and soldered to the pads of ZIF connectors using silver epoxy (Epotek E4110). The connection was encapsulated in epoxy resin and the samples were released and placed in phosphate-buffered saline at 37 °C, while the resistance values were measured with a data logger switching unit (Keysight 34970 A).

### Temperature coefficient of resistance measurements

Zn resistors were patterned by screen printing and sintered using the two-step process presented above. The resistance was monitored continuously with a digital multimeter (Keysight 34401 A), and reference temperature was acquired using a commercial sensor (Sensirion SHT4x). The temperature was varied in a custom-made chamber (or a plastic vial in the case of experiments conducted in PBS) with the help of a hot plate.

### Pressure sensors fabrication and characterization

The different designs for the pressure sensors were printed with a laser-cut stencil and treated with the aforementioned sintering process. POMaC was synthesized as previously described. Maleic anhydride, citric acid and 1,8 octanediol (all from Sigma-Aldrich) were mixed at a molar ratio of 3:2:5 in a three-necked flask. The mixture was heated to 160 °C (under nitrogen flow and 200 rpm stirring) until the reagents were fully melted and then reacted at 140 °C for 3 h. The pre-polymer was dissolved in THF and purified by drop-wise purification in DI water, decanted, and dried. The pre-polymer was mixed with 5 wt% of the photoinitiator Irgacure 2959. The POMaC pre-polymer/photoinitiator mixture was then blade-cast with a gap of 500 µm and a speed of 3 mm s^−1^ and cured under UV (Proma 140001, 60 W, 365 nm) for 20 min. The outlines of the sensors were then cut with a laser engraver (Trotec Speedy 300), they were then gently peeled from the silicon wafer, folded, and cured at 80 °C for 48 h. Concerning the sensor behavior, uniaxial pressures were applied with a pull-tester (Instron 3340) and the capacitance values were recorded using an LCR-meter (E4980A) at 2 MHz. For reference, the Young’s modulus of the POMaC was measured with a standard stress-strain test (ASTM D412) and the relative permittivity was estimated by measuring the capacitance of POMaC layers in a parallel plate capacitor configuration with sputtered gold electrodes. In the case of the wireless Zn sensors, the S11 coefficient was measured with a vector network analyzer (NanoVNA V2) with the aid of a custom-made reader coil (silver on FR4). The resonant frequency of the sensor was extracted with a custom algorithm written in python.

### Wirelessly powered circuit

A similar fabrication process to the one described above for the wireless pressure sensors was used. POMaC was used as a bonding layer and was separately blade-cast on a silicon wafer with a polyacrylic acid sacrificial layer. The POMaC was cured under UV, laser-cut, released in DI water, and laminated on the bottom half of the PLA coil. The top half was limited onto the POMaC layer after filling the vias with silver epoxy, and the assembled sensor was cured at 80 °C for 48 h to allow the POMaC to fully polymerize. Finally, the SMD components (green LED and 470 pF capacitor) were connected to the Zn lines using the Zn ink described above mixed with 10 vol% acetic acid.

### Supplementary information

Supplementary Figures

## Data Availability

The data that support the findings of this work are available from the corresponding author upon reasonable request.
