# Supplementary material for: Zinc hybrid sintering for printed transient sensors and wireless electronics
Source: Npj Flex Electron. 2023 Mar 14;7(1):14. doi: 10.1038/s41528-023-00249-0 (PMC11041761; doi:10.1038/s41528-023-00249-0)
Supplement: Supplementary file 1 — Supplementary Figures [file 41528_2023_249_MOESM1_ESM.pdf]

Supplementary Figures

Zinc Hybrid Sintering for Printed Transient Sensors and Wireless Electronics

Nicolas Fumeaux and Danick Briand**

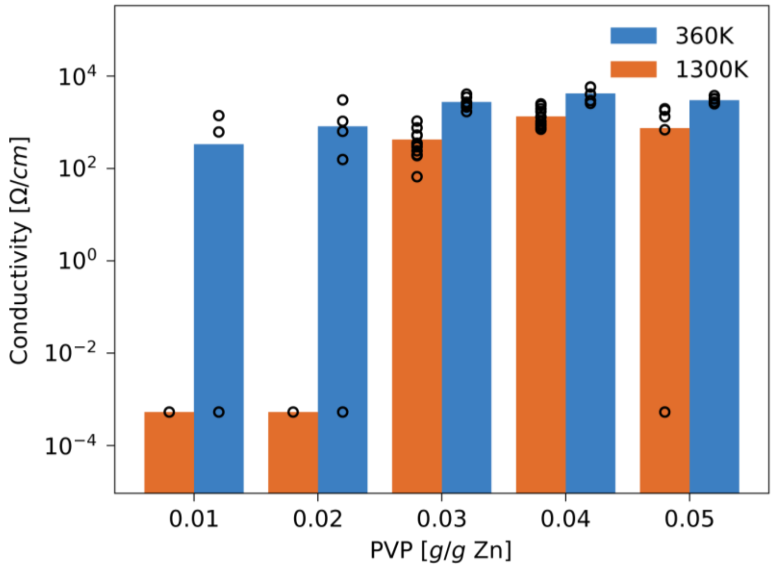

Supplementary Figure 1. Electrical conductivity of lines treated by electrochemical acetic acid sintering as a function of the weight of PVP per weight of Zinc in the solution.

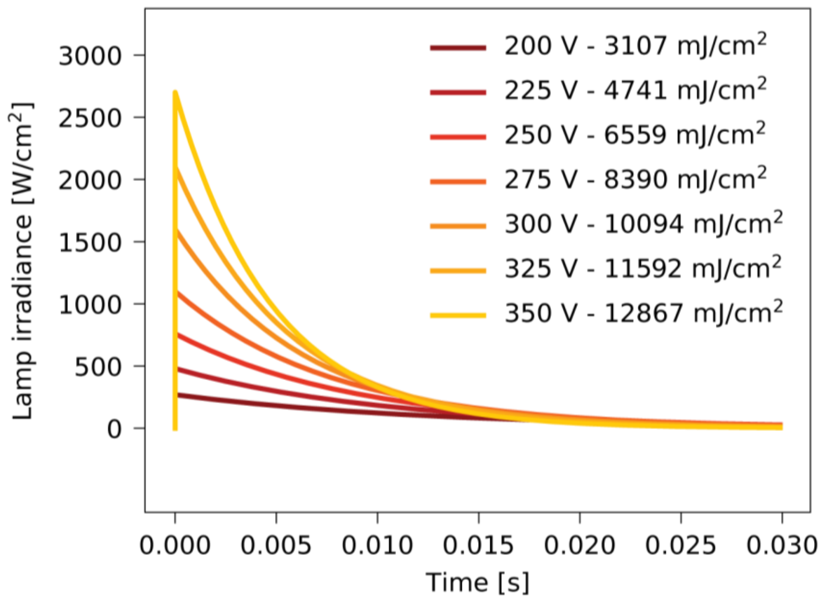

Supplementary Figure 2. Pulses delivered by the PulseForge 1200 photonic sintering lamp as a function of lamp voltage, given as irradiance during the pulse delivery time.

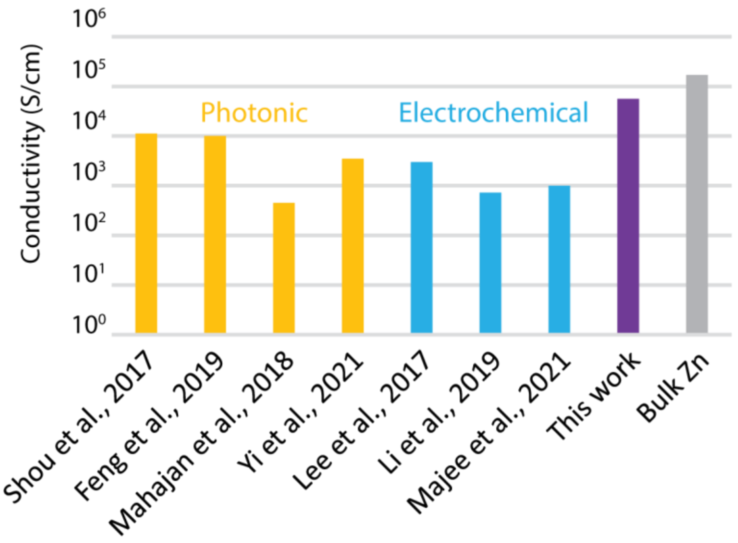

Supplementary Figure 3. Electrical conductivity of the hybrid sintered Zn traces from this work compared to the conductivity of bulk zinc and to examples from the literature for printed zinc using different sintering methods.

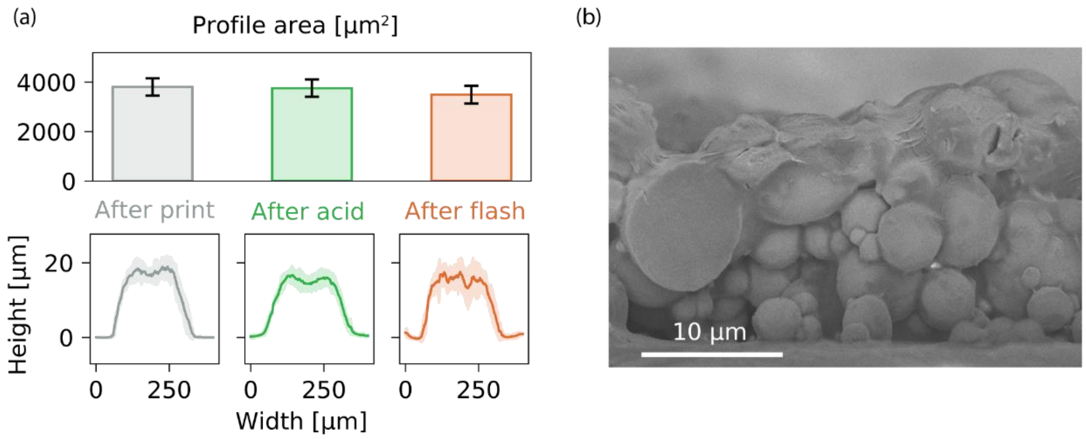

Supplementary Figure 4. Study on profile geometry and microstructure for sintered screen-printed traces. (a) Area and average height of screen-printed profiles after printing, acetic acid sintering, and photonic sintering. (b) SEM cross-section of sintered Zn on polyimide, after freezing in liquid nitrogen and cutting. The sintering and agglomeration of the particles are gradual and localized, particularly in the top 5-10 µm.

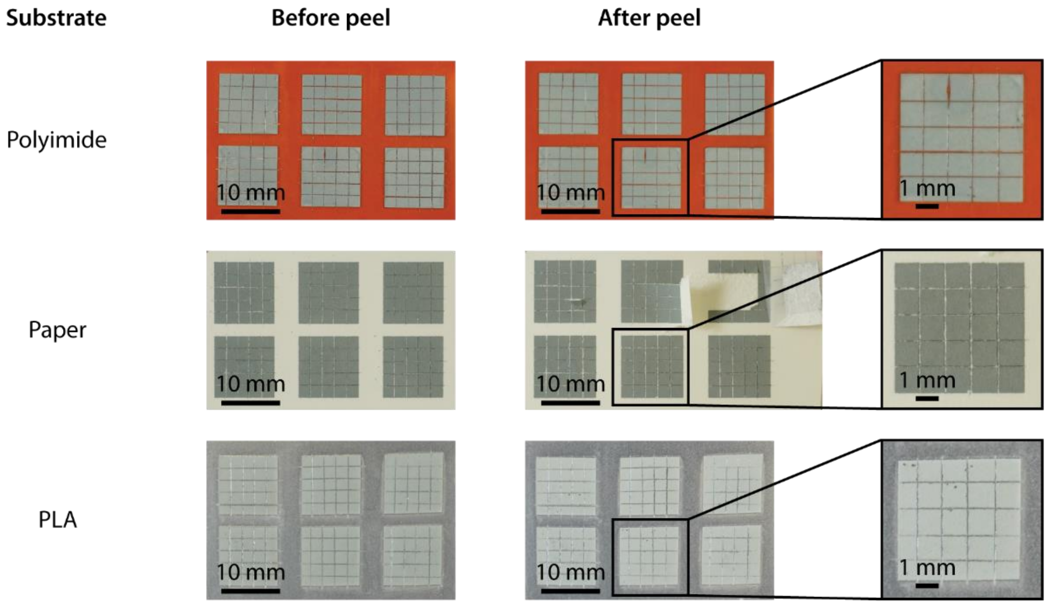

Supplementary Figure 5. Peel test according to ASTM F1842-15 on polyimide, paper and polylactic acid substrates. The sintered zinc layer demonstrated good adhesion properties with the substrates with a classification ranging from 5B to 4B. In the case of paper, the surface part of the substrate peeled during the test, and the zinc layer remained attached to the paper.

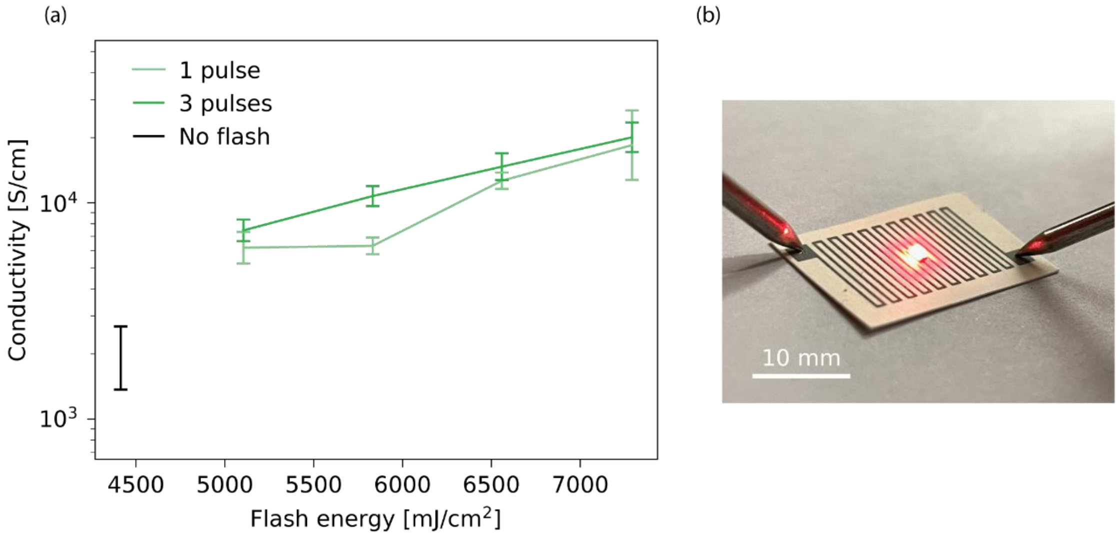

Supplementary Figure 6. Hybrid sintering on paper. (a) Conductivity measured from printed Zn resistors on paper after hybrid sintering, as a function of flash energy and number of pulses. (b) Zn tracks on paper connected to an LED.

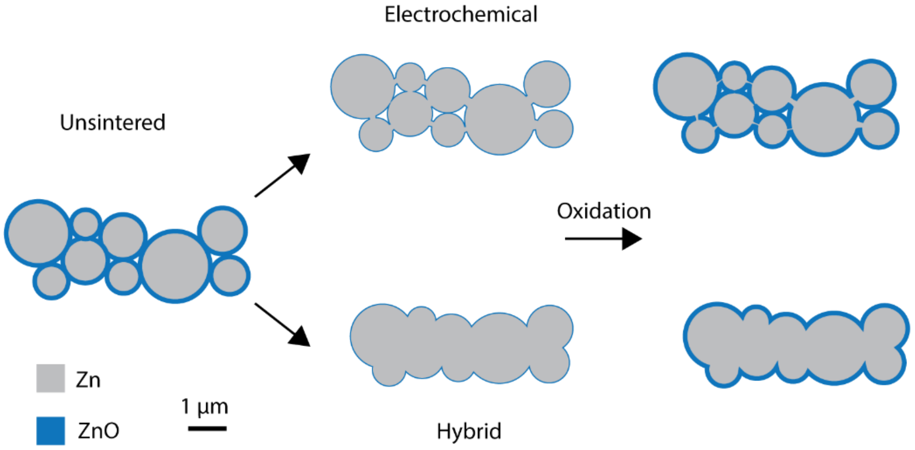

Supplementary Figure 7. Proposed mechanism for the longer durability of photonic sintered Zn in air. The enhanced particle cohesion obtained with hybrid sintering decreases the impact of the zinc reoxidation on the electrical conductivity.

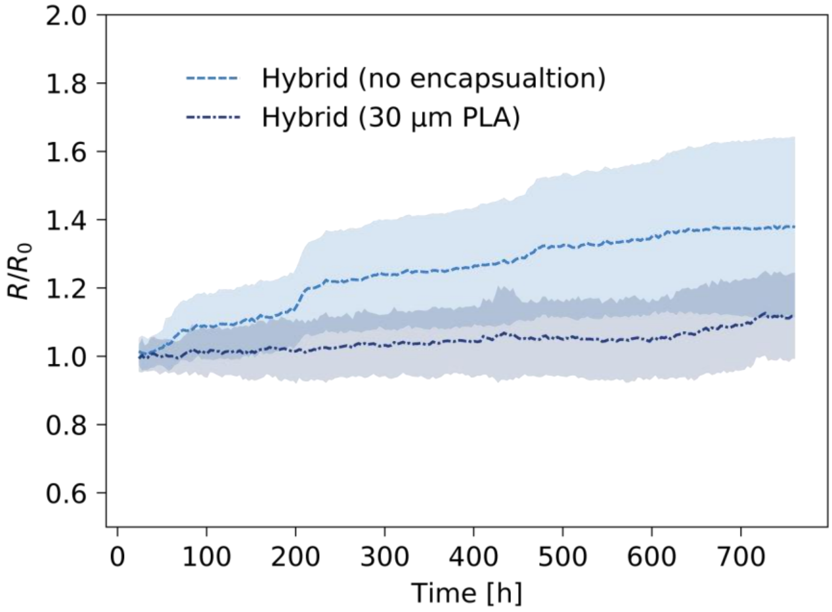

Supplementary Figure 8. Relative change of resistance for Zn lines on PLA after hybrid sintering in air over a period of 31 days, with and without encapsulation.

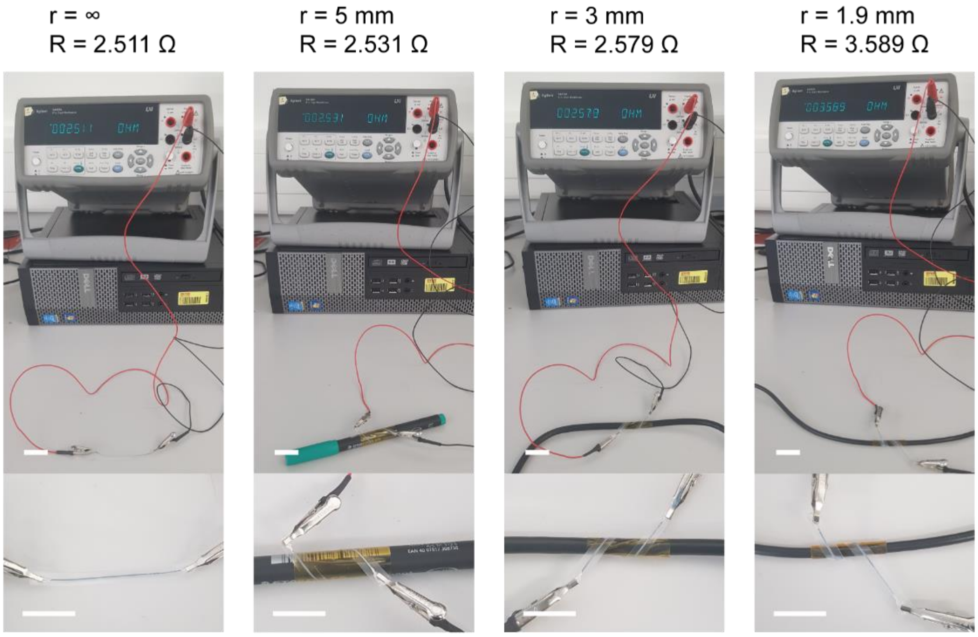

Supplementary Figure 9. Bending of a printed and encapsulated Zn resistor on PLA (50 × 0.5 mm) at different bending radii. All scale bars are 20 mm.

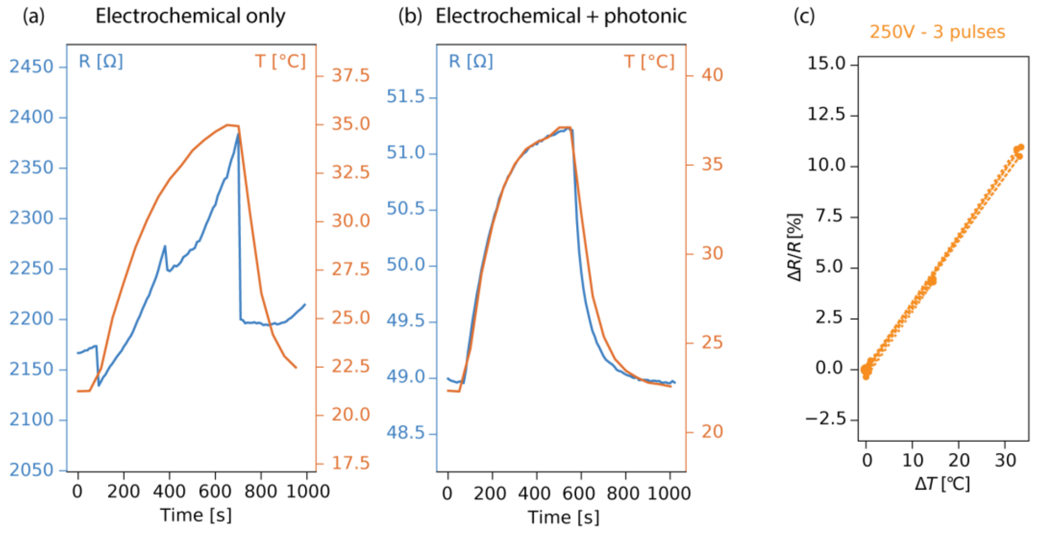

Supplementary Figure 10. Temperature behavior of Zn lines as a function of the sintering process. (a) Heating and cooling of acid sintered only Zn resistor. (b) Heating and cooling of hybrid sintered Zn resistor. (c) Change of resistance with respect to difference from room temperature for a lamp voltage of 250 V and 3 pulses applied.

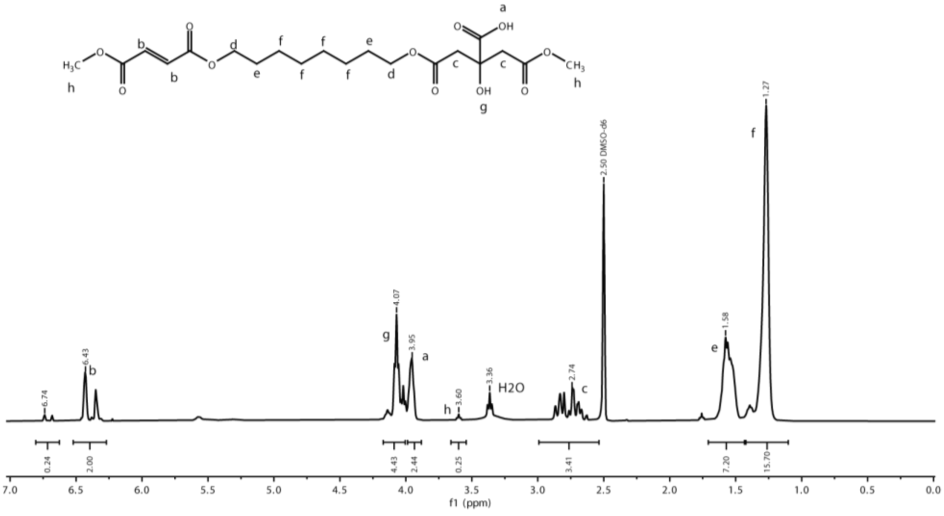

Supplementary Figure 11. ^{1}H-NMR for the synthetized POMaC pre-polymer. ^{1}H NMR spectra were recorded on a Bruker AVANCE-400 Ultra Shield instrument at room temperature using $(CD_3)_2SO$ as solvent. ^{1}H NMR chemical shifts are reported relative to the residual proton signal of the solvent.

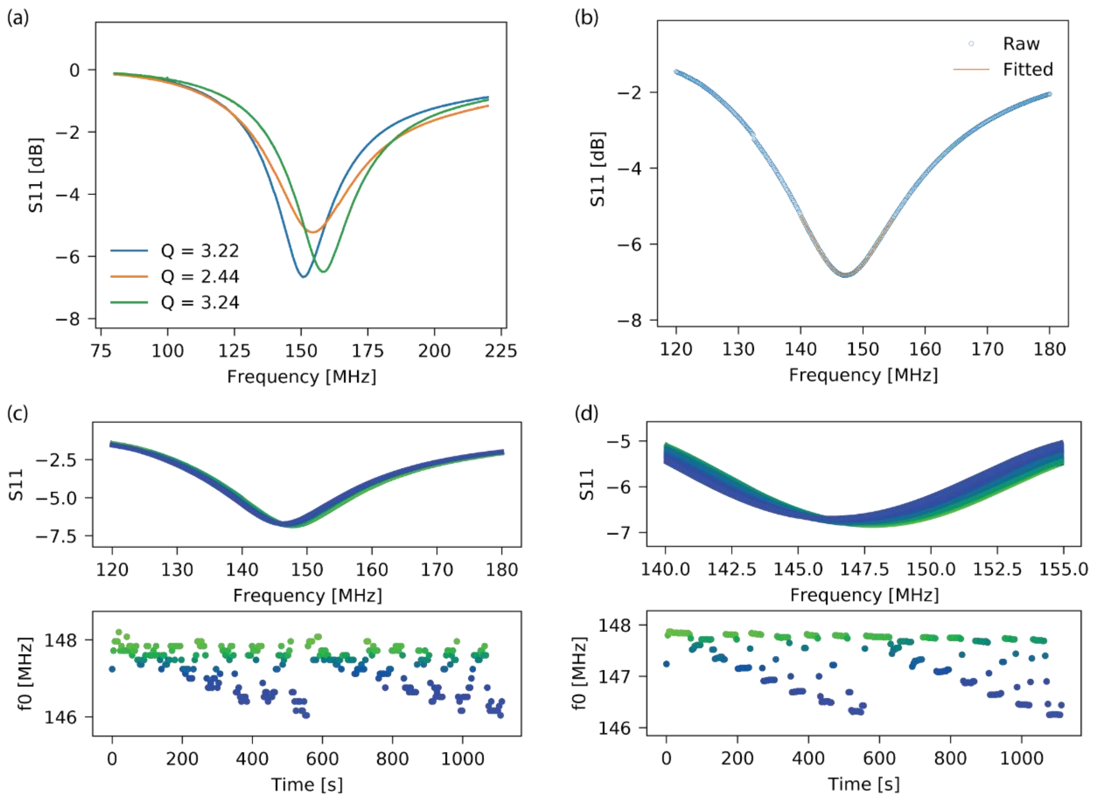

Supplementary Figure 12. Real-time data processing for the S_{11} data of pressure-sensitive wireless circuits. (a) Return loss (S_{11}) of typical transient wireless pressure sensors. (b) 5th-degree polynomial fit used to smooth the resonant frequency signal. (c) The S_{11} signal and corresponding resonant frequency during application of a calibration force signal, without polynomial fit smoothing. (d) The same data with the polynomial fit smoothing.

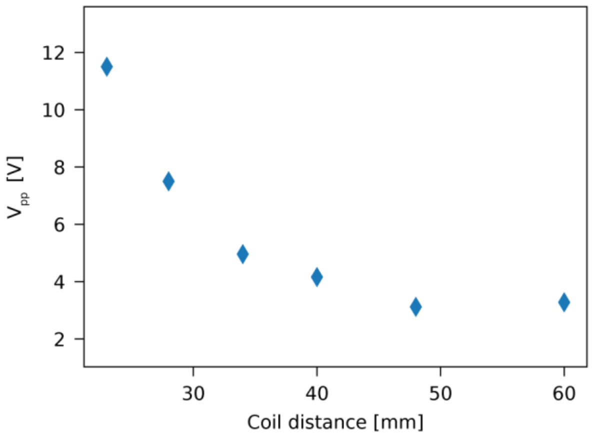

Supplementary Figure 13. Peak-to-peak recorded voltage at the secondary coil (degradable power receiver) as a function of the distance to the primary coil.
